# Targeted Degradation of sGRP78 Alleviates the Immunosuppressive Tumor Microenvironment

**DOI:** 10.1002/advs.202509921

**Published:** 2025-09-11

**Authors:** Zhenghao Wu, Peng Zheng, Yunxiao Xiao, Qianheng Wang, Xin Pan, Xiaoqi Zhou, Yibing Lv, Junyi Xiao, Yong He, Tao Huang, Ping Lei

**Affiliations:** ^1^ Department of Immunology, School of Basic Medicine, Tongji Medical College and State Key Laboratory for Diagnosis and Treatment of Severe Zoonotic Infectious Diseases Huazhong University of Science and Technology Wuhan 430030 China; ^2^ Department of Breast and Thyroid Surgery Union Hospital, Tongji Medical College, Huazhong University of Science and Technology Wuhan 430022 China; ^3^ Department of Nuclear Medicine Zhongnan Hospital Wuhan University Wuhan 430071 China

**Keywords:** breast cancer, glucose‐regulated protein 78, regulatory B cells, targeted protein degradation, tumor immunity

## Abstract

There is an urgent need for appropriate methods to identify patients who are sensitive to neoadjuvant therapy (NAT) for precise stratified treatment. Based on clinical proteomics screening and further validation in patient cohorts, we reported that soluble glucose‐regulatory protein 78 (sGRP78) could be a serological biomarker to infer the poor chemotherapy response in breast cancer. Consequently, an “sGRP78 index” was established to accurately predict patient responses to NAT. When exploring the linked mechanisms on how sGRP78 confer the resistance, it was found that in high sGRP78 index tumors, there were more IL‐10+/PD‐L1+ B subsets and Tregs infiltration, accompanied by accelerated tumor progression and metastasis. The following experiment revealed that sGRP78 bound with tumor‐infiltrating B cells, converting the latter into IL‐10+/PD‐L1+ ones, thereby promoting Treg formation and suppressing T cell‐mediated antitumor cytotoxicity. By fusing the GRP78‐selective protease subtilase cytotoxin catalytic A subunit (subA) with a nanobody against HER2, we achieved the targeted degradation of sGRP78 within the breast cancer region, effectively reversing the immunosuppressive microenvironment. Our findings highlight the potential of sGRP78 index as a predictive signature to identify patients’ sensitivity to NAT, as well as the potential of sGRP78 as a novel immune checkpoint target for cancer therapy.

## Graphical abstract

Soluble GRP78 (sGRP78) is identified as a novel tumor‐derived soluble factor that induces regulatory T cells (Treg). sGRP78 serves as an excellent serological biomarker for predicting the efficacy of neoadjuvant therapy in breast cancer patients. sGRP78 specifically binds to B cells, inducing the expression of IL‐10 and PD‐L1, thereby promoting Treg cell differentiation. Nanobody‐subA fusions enable targeted degradation of sGRP78, effectively reversing sGRP78‐mediated immune evasion.

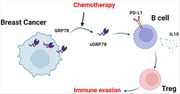

## 1 Introduction

Breast cancer (BC) continues to be one of the leading causes of tumor‐related death in women, seriously threatening women's health.^[^ ^1^ ^]^ Chemotherapy is still one of the standard treatment methods for BC patients. However, even with the best chemotherapy regimen available today, only 39% of patients who have undergone neoadjuvant chemotherapy (NAT) achieve pathological complete response (pCR). Chemotherapy‐resistant tumor cells appear in up to 60% of patients after six months course.^[^ ^2^ ^]^ Therefore, there is an urgent need for appropriate methods to identify patients who are sensitive to chemotherapy for precise stratified treatment. There are now several predictive gene signatures used in clinical practice, such as Oncotype DX and the MammaPrint score.^[^ ^3^, ^4^ ^]^ However, the predictive value of these panels for triple‐negative breast cancer (TNBC) and HER2‐positive breast cancer has not been well supported by evidence, suggesting that more biomarkers are yet to be discovered.

Glucose regulated protein 78 (GRP78, also referred to as BiP), a member of the highly conserved HSP70 family, is a central regulator of endoplasmic reticulum homeostasis by playing key roles in nascent protein chain folding, transporting, and quality control. This ER‐resident chaperone is upregulated under stress conditions, including hypoxia, nutrient deprivation, virus infection, and others, to afford cytoprotection.^[^ ^5^ ^]^ Adapted to these chronic stresses in the tumor microenvironment, cancer cells are observed to upregulate the expression of GRP78 to promote their proliferation, invasion, resistance to treatment, and immune escape, making GRP78 a typical oncogene.^[^ ^5^ ^]^ The upregulation leads GRP78 to escape from the ER and translocate to the extracellular fluids.^[^ ^6^ ^]^ The soluble form of GRP78 has been widely accepted as an immunoregulatory molecule to favor the resolution of immune response ^[^ ^7^ ^]^ by generation of regulatory T‐cell ^[^ ^8^ ^]^ and B‐cell ^[^ ^9^ ^]^ population, by affecting maturation of dendritic cells ^[^ ^10^ ^]^ and by impairing production of proinflammatory cytokines.^[^ ^11^ ^]^ GRP78 release could infer the intensity of chemo‐related damage.^[^ ^12^, ^13^ ^]^

In this study, based on clinical and experimental data, we reported that sGRP78 could be a serological biomarker to infer the poor chemotherapy response in breast cancer, and the “sGRP78 index” was a predictive signature to identify patients’ sensitivity to NAT for precise stratified treatment. sGRP78 is a potential immune checkpoint molecule, and its targeted degradation could alleviate the immunosuppressive tumor microenvironment to facilitate tumor therapy.

## 2 Results

### 2.1 sGRP78 Index as a Serum Biomarker for Patient's Response to Chemotherapy

**Figure 1:**
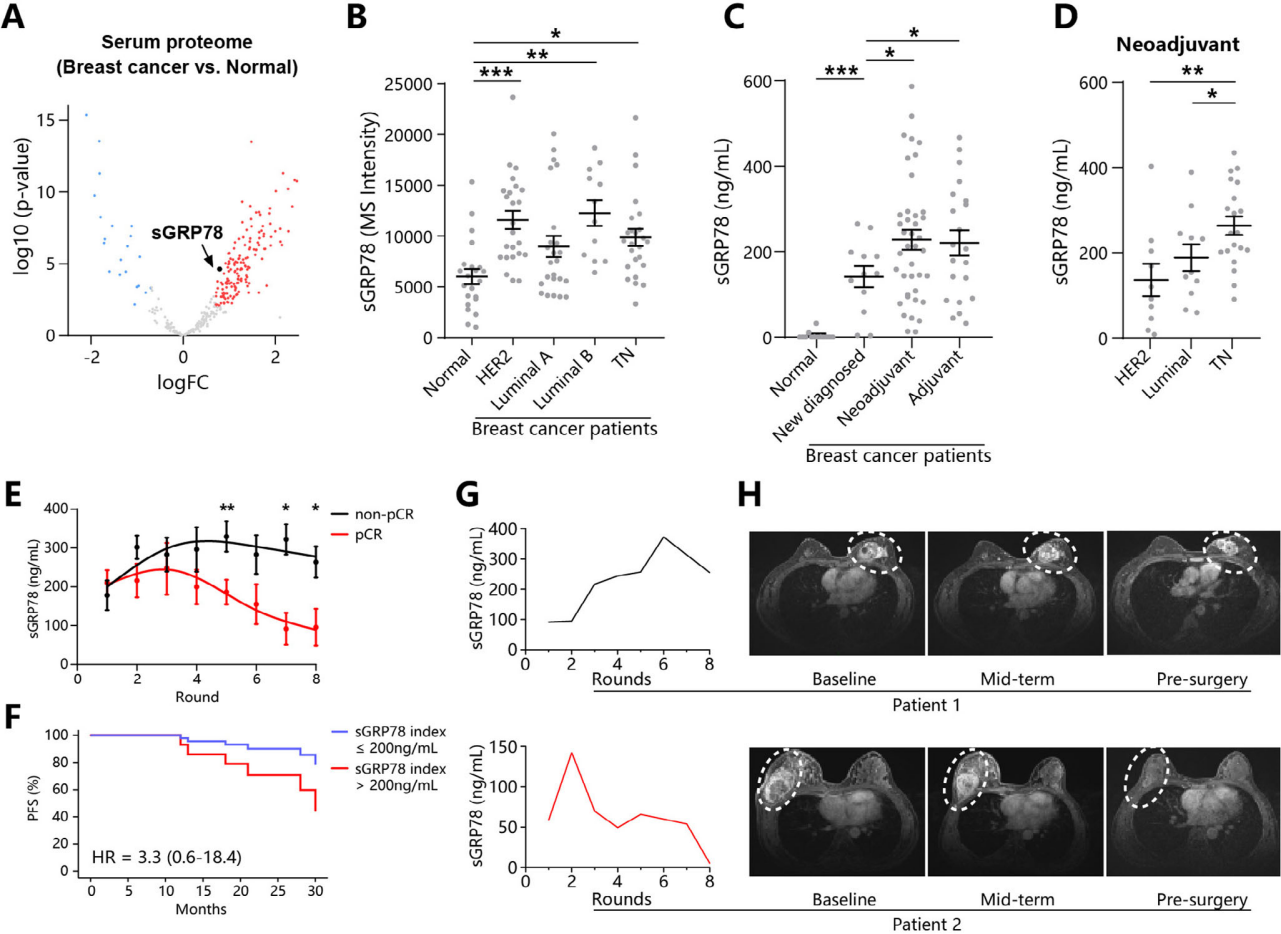
sGRP78 level is associated with poor chemotherapy response in breast cancer A) Volcano plot of the differentially expressed serum proteins in breast cancer patients and healthy volunteers, as determined by MS‐based proteomic technology. Proteins were upregulated (red; *p* < 0.05, FC >2) or downregulated (green; *p* < 0.05, FC←2). Data are presented as mean ± SEM of *n* = 76. B) MS intensity of serum sGRP78 in breast cancer patients with four different molecular subtypes (luminal A/B, HER2, TN). Serum sGRP78 concentration in breast cancer patients C) when they were newly diagnosed, during neoadjuvant or adjuvant therapy, or D) with different subtypes during NAT. For each patient, sGRP78 concentration was calculated as the average value for multiple rounds of chemotherapy. E) The dynamic changes of serum sGRP78 in patients receiving NAT. Patients were divided into pathological complete remission (pCR) and non‐pCR groups according to pathological results. F) Kaplan‐Meier estimates of PFS for patients receiving NAT, stratified by sGRP78 index. G) sGRP78 levels in each round of therapy and H) MRI pictures of typical non‐pCR (upper) and pCR (lower) patients. ^*^ *p* < 0.05, ^**^ *p* < 0.01, ^***^ *p* < 0.001. Data are presented as mean ± SEM of *n* = 43.

To explore new serum biomarkers for breast cancer, a serum proteomic dataset of newly diagnosed breast cancer and normal volunteers^[^ ^14^ ^]^ was analyzed. This dataset included 76 breast cancer patients who had not received any treatment, and excluded patients with chronic inflammation, hypertension, and other history of malignancy. Our analysis showed that sGRP78 was significantly enriched in the serum of breast cancer patients compared with healthy volunteers (**Figure** **1**A). In the four molecular subtypes of breast cancer, sGRP78 levels were all evidently increased, especially in HER2‐positive, Luminal B and triple‐negative (TNBC) types (Figure 1B), suggesting a potential role of sGRP78 as a serum biomarker to diagnose breast cancer. Our prospective study also confirmed that serum sGRP78 concentration was much higher in newly diagnosed patients than in healthy controls (Figure 1C). Notably, its level was further elevated and exceeded 200 ng mL^−1^ in patients receiving chemotherapy (Figure 1C), and reached the highest point in TNBC patients under neoadjuvant therapy (Figure 1D).

Considering the regulatory role of sGRP78 in immune response and inflammation,^[^ ^10^, ^11^, ^15^, ^16^, ^17^ ^]^ to accurately measure the influence of chemotherapy‐induced sGRP78 on patients’ response to therapy, a cohort including 43 breast cancer patients who underwent NAT (as summarized in Table S1, Supporting Information) was established. Data showed that serum sGRP78 concentration rapidly increased in patients after the first round of therapy, peaked at round 3 with a mean concentration around 264 ng mL^−1^. It then gradually decreased and reached a nadir at around 7 with a slight increase afterwards (Figure S1A, Supporting Information). After mastectomy, according to their pathological results, patients were grouped as pathological complete remission (pCR) and non‐pCR (npCR). It was observed that although sGRP78 concentration had no difference in the first four rounds between both groups, its level in npCR patients increased steadily and remained above 200 ng mL^−1^, while in pCR patients fluctuated around 200 ng mL^−1^. In the latter four rounds, sGRP78 decreased evidently in the pCR group, compared to a slight decline from peak concentration in npCR patients (Figure 1E). These results indicated that chemotherapy‐induced sGRP78 was associated with poor therapy response.

sGRP78 concentration had no difference in the first three rounds between both groups. The level started to decrease in the fourth round in the pCR group, while in npCR patients, sGRP78 concentration peaked at the 4th round with a slight decrease in the latter four rounds (Figure 1E). Therefore, sGRP78 levels measured at the 4th round can serve as an earlier discriminative biomarker for predicting neoadjuvant therapeutic response in breast cancer patients. And accordingly, “sGRP78 index” was proposed for monitoring patients’ response during NAT, defined as the serum sGRP78 levels after the 4th round of chemotherapy. Both univariate and multivariate logistic regression analysis revealed low sGRP78 index as an independent predictor of pCR after NAT (Table S2, Supporting Information), supporting its potential prognostic utility. The receiver operating characteristic (ROC) curve demonstrated that the sGRP78 index reliably predicted pathological complete response (pCR) (Figure S1B, Supporting Information). For choosing the cut‐off that maximized the Youden index, we set the threshold at 200 ng mL^−1^, corresponding to a sensitivity of 0.78 and a specificity of 0.67. Patients with “sGRP78 index > 200 ng/mL” had a markedly reduced progression‐free survival (PFS) compared to those with low sGRP78 index [HR, 3.3; 95% confidence interval (CI), 0.6 to 18.4; *p* = 0.172] (Figure 1F). Kaplan–Meier estimates of PFS at 2 years were 70% for the “sGRP78 index > 200 ng mL^−1^” and 90% for the “sGRP78 index ≤ 200 ng mL^−1^” groups. Therefore, sGRP78 index potentially predicts the response of breast cancer patients after NAT.

As shown in typical patient 1, her sGRP78 index was 243.7 ng mL^−1^. It could be observed that her serum sGRP78 gradually increased and peaked on the sixth round of chemotherapy, with >3 times higher than the initial value. There was also maintained a large mass in the breast, and the postoperative pathology showed Miller‐Payne (MP) grade G2 (the reduction of tumor cells does not exceed 30%). The primary lesion also recurred after completion of the adjunctive therapy. For patient 2 (sGRP78 index = 49.5 ng mL^−1^), sGRP78 experienced a short‐term increase and then gradually declined to a very low level. Correspondingly, the patient's primary tumor rapidly shrank with pCR confirmed. No recurrence or metastasis was found during two years of follow‐up (Figure 1G,H). Notably, the mid‐term MRI scan of both patients was not enough to indicate the chemotherapy response, while the “sGRP78 index” was already available and differentiated. Therefore, we provide a potential sensitive and non‐invasive serum biomarker, sGRP78 index, to monitor the efficacy of NAT.

Then we evaluated the relationship between sGRP78 and other tumor attributes. Serum sGRP78 level before any treatment was positively correlated with the TNM stage of cancer patients (Figure S1C, Supporting Information). Patients with large tumor diameter and lymph node metastasis had significantly higher sGRP78 level, while with distant metastasis did not (*P* = 0.13). Hence, baseline sGRP78 will be higher in advanced patients (Figure S1C, Supporting Information). Given that advanced TNBC is more likely to be insensitive to chemotherapy, TNM stage might be the confounding factors between sGRP78 and chemotherapy resistance. However, our data manifested that average sGRP78 levels during every round of NAT were irrelevant to the TNM stage, suggesting chemotherapy blurs the difference of serum sGRP78 among tumor burden and metastasis (Figure S1D, Supporting Information).

In summary, sGRP78 is strongly relevant to chemotherapy resistance in breast cancer patients.

### 2.2 Chemotherapy Induces Breast Cancer Cells to Release sGRP78

**Figure 2:**
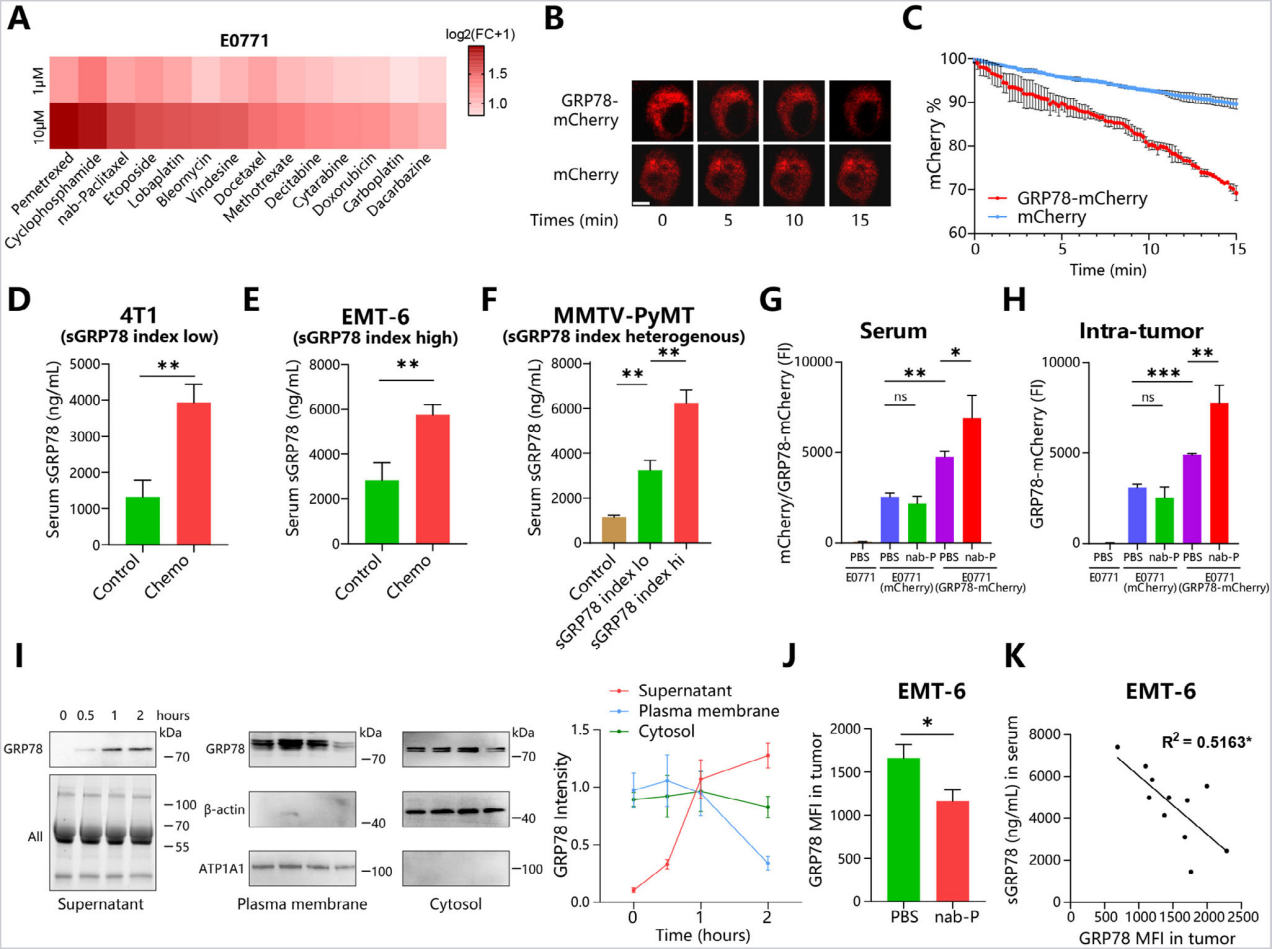
Chemotherapy induces cancer cells to release sGRP78 A) Fold change (FC) of GRP78‐mCherry fluorescence intensity in the supernatant of E0771(GRP78‐mCherry) cells treated with chemotherapeutic drugs (1 or 10 µm) for 48 h. B,C) The chamber containing E0771 cells was mounted onto the microscope stage. After the supplement of nab‐P (10 µm), images of mCherry fluorescence were acquired continuously at 10 s intervals for 15 min. Presented were the selected ones taken during this period. B) Images were indicated by time points, Scale bar = 5 µm, and C) dynamic changes in the fluorescence intensity were shown. The dynamic change of mCherry MFI was calculated compared with baseline levels. Serum concentration of sGRP78 in (D) 4T1 tumor bearing mice treated with Lb (10 mg kg^−1^ i.p. q2d, *n* = 8), E) EMT‐6 tumor bearing mice with nab‐P (10 mg kg^−1^ i.p. q2d, *n* = 8), and F) MMTV‐PyMT mice treated with nab‐P (20 mg kg^−1^ i.p. q1w, *n* = 8). Fluorescence intensity of sGRP78‐mCherry in G) serum and H) tumor interstitial fluid of E0771 tumor bearing mice treated with nab‐P. I) Western blot for GRP78 in different subcellular locations of MDA‐MB‐231 cells at multiple time points after nab‐P treatment. Representative blot images (left) and dynamic GRP78 changes (right) were shown. J) Expression of membrane bound GRP78 on tumor cells isolated from Lb‐treated EMT‐6 mice model. K) Correlation analysis between the intensity of tumor GRP78‐mCherry and serum sGRP78 level in mice receiving chemotherapy. ^*^ *p* < 0.05, ^**^ *p* < 0.01, ^***^ *p* < 0.001. q1w: once per week; q2d: once per two days; i.p.: intraperitoneal injection.

To evaluate the sGRP78‐releasing capacity induced by various therapeutics, the breast cancer cell line E0771 was engineered to express GRP78‐mCherry (mCherry was fused to GRP78 via a flexible linker to prevent interference with GRP78 function, as shown in Figure S2A, Supporting Information) and then exposed to chemotherapeutics. GRP78‐mCherry fluorescent intensity in the supernatant was measured as a readout for sGRP78 release. Results in **Figure** **2**A showed that nearly all chemotherapy drugs promoted the release of sGRP78 from E0771(GRP78‐mCherry) cells, as well as from melanoma B16‐F10(GRP78‐mCherry) cells (Figure S2B, Supporting Information), indicating chemotherapy‐induced sGRP78 release was a common phenomenon. However, the capacity of E0771 to release sGRP78 was relatively weaker (Fold change: 1–2) than that of B16‐F10 (Fold change: 1–6). In addition, these two cell lines were sensitive to quite different drugs. E0771 was sensitive to nab‐paclitaxel, cyclophosphamide, and lobaplatin, all of which are commonly used for breast cancer. To accurately monitor sGRP78 release, nab‐P treated E0771 cells were dynamically detected for their mCherry intensity. Using the loss of cellular fluorescence as a readout for sGRP78 release (Figure 2B), we showed that fluorescence intensity in GRP78‐mCherry group decreased more rapidly than in mCherry group (2.05%/min versus 0.69%/min, Figure 2C). E0771(GRP78‐mCherry) cells maintained the high‐speed decrease of cellular fluorescence within 15 min, and even had a tendency to accelerate gradually (Figure 2C). Therefore, chemotherapeutic drugs induce sGRP78 release quickly and massively in vitro.

To verify the sGRP78 release in vivo, three chemotherapy‐treated breast cancer models were established: 1. nab‐P treated EMT‐6 tumor bearing Balb/c mice; 2. lobaplatin (Lb)‐treated 4T1 tumor bearing Balb/c mice; 3. nab‐P treated transgenic MMTV‐PyMT spontaneous breast cancer model. Serum was collected after the 4th round of chemotherapy, consistent with the criteria for calculating the sGRP78 index in patients. It was observed that the concentrations of serum sGRP78 in the chemotherapy groups were significantly higher than those in the control mice (Figure 2D–F). When comparing the distribution of serum sGRP78 levels in humans and mice after chemotherapy, the sGRP78 index threshold for the murine model was set at 4400 ng mL^−1^ (22 times that of humans). Based on this, the 4T1 was identified as low while EMT‐6 was identified as a high sGRP78‐releasing cell line, respectively (Figure 2D,E). The MMTV‐PyMT transgenic mouse, being a spontaneous tumorigenesis model driven by oncogenes, exhibits significant individual heterogeneity as per prior studies.^[^ ^18^, ^19^, ^20^ ^]^ Accordingly, notable differences in serum sGRP78 levels among different MMTV‐PyMT mice were observed. Based on the 4400 ng/mL threshold, MMTV‐PyMT mice were categorized into sGRP78‐releasing high and low groups (Figure 2F). Correspondingly, E0771(GRP78‐mCherry) tumor bearing mice exhibited the release of fluorescent tagged GRP78 in serum and in tumor tissues after chemotherapy (Figure 2G,H).

Above data suggested chemotherapy directly induced 2–3 times more sGRP78 release in vivo, regardless of cancer models and types of drugs, in line with our clinical data (Figure S1A, Supporting Information). Furthermore, tumor‐derived GRP78 (GRP78‐mCherry measured by fluorescence spectrophotometer) was positively correlated with total sGRP78 (measured by ELISA), showing that the tumor was the major source of serum sGRP78 in these models (Figure S2C, Supporting Information).

Found in the ER, plasma membrane, and cytoplasm in cells, GRP78 can theoretically be released through ER‐Golgi, plasma membrane‐derived microparticles, and exosome secretory routes. To clarify the route of secretion in breast cancer cells, GRP78 was evaluated in different cellular compartments in drug‐treated cells. In MDA‐MB‐231 cells, as sGRP78 concentration gradually increased in the supernatant within 2 h of exposure to nab‐P, cell surface GRP78 decreased rapidly, with intracellular GRP78 remaining stable (Figure 2I). In EMT‐6 tumor bearing mice, membranous GRP78 expression on cancer cells (CD45^−^EpCAM^+^) significantly reduced after therapy (Figure 2J), and negatively correlated with serum sGRP78 concentration (Figure 2K). These data confirmed the secretion of GRP78 by breast cancer cells and provided evidence that GRP78 could be shed from the cell membrane and released a soluble form after chemotherapy.

### 2.3 sGRP78 Release Promotes Cancer Drug Resistance

**Figure 3:**
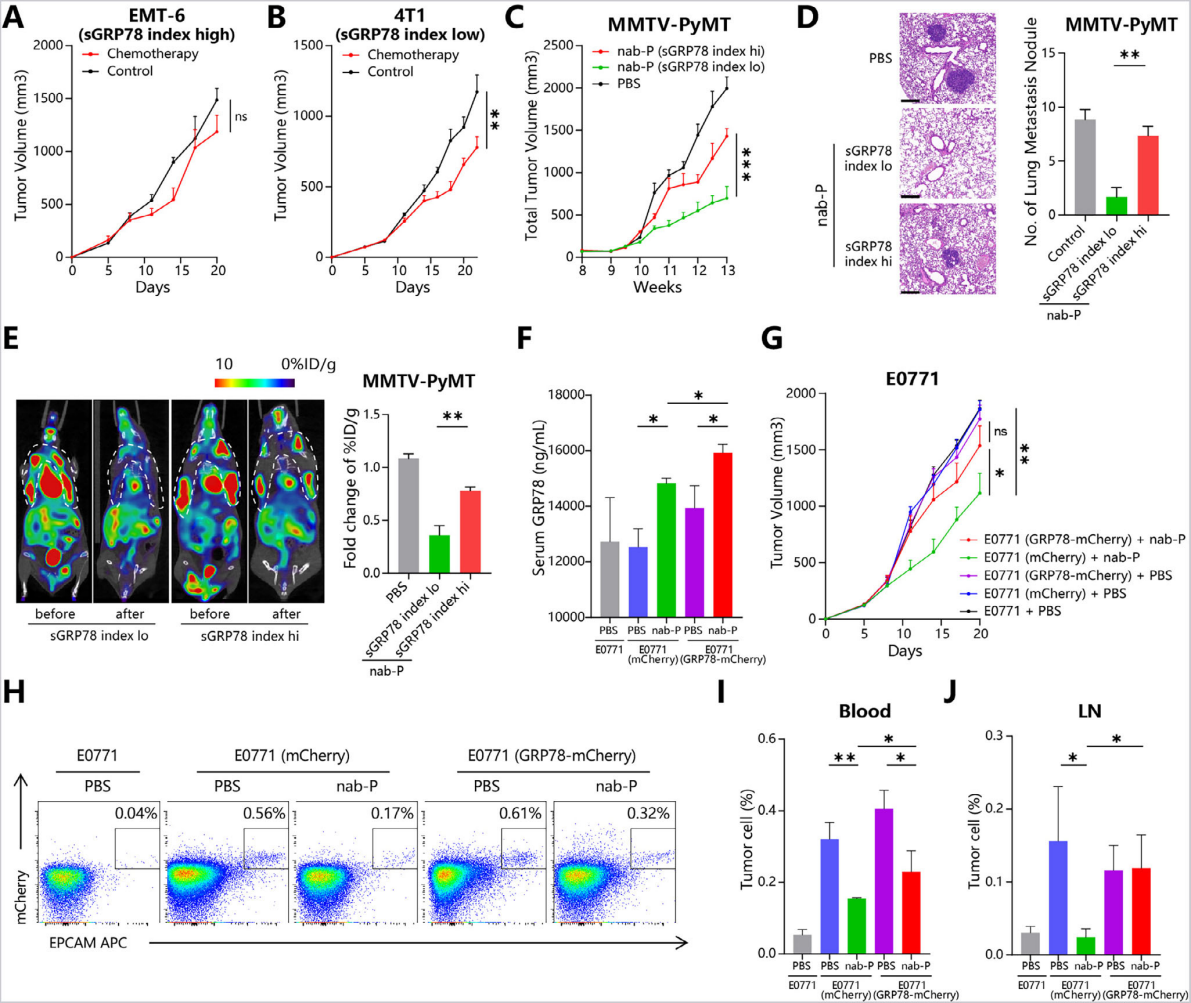
sGRP78 promotes cancer resistance to chemotherapy. Tumor volumes in A) EMT‐6 tumor bearing mice with nab‐P (10 mg kg^−1^ i.p. q2d;*n* = 8), B) 4T1 tumor bearing mice treated with Lb (10 mg kg^−1^ i.p. q2d;*n* = 8), and C) MMTV‐PyMT mice treated with nab‐P (20 mg kg^−1^ i.p. q1w;*n* = 8). C–E) MMTV‐PyMT mice were dichotomously grouped according to their sGRP78 index with 4200 ng mL^−1^ as the threshold. D) Lung metastasis in MMTV‐PyMT mice. Representative HE staining (left) and numbers of metastasis nodules (right) were showed, scale bar = 200 µm. E) 18F‐FDG PET/CT imaging for MMTV‐PyMT mice before and after chemotherapy, with primary breast cancer lesions in the white dotted line. Representative images (left) and fold change of 18F‐FDG uptake in lesions (right) before and after chemotherapy were shown. Serum sGRP78 concentration F) and tumor volume G) in E0771 (wildtype, mCherry or GRP8‐mCherry) tumor bearing mice with nab‐P (10 mg kg^−1^ i.p. q2d;*n* = 8). The frequencies of CD45‐mCherry+EpCAM+ tumor cells in H–I) peripheral blood and J) lymph nodes were identified by FCM. ^*^ *p* < 0.05, ^**^ *p* < 0.01, ^***^ *p* < 0.001. q1w: once per week; q2d: once per two days; i.p.: intraperitoneal injection.

Breast cancer patients with a higher sGRP78 index have poorer response to NAT (Figure 1E,F). To test this phenomenon in murine models, the sGRP78^hi^ and sGRP78^lo^ tumor‐bearing models were established as described above. It was found that in mice bearing with a high sGRP78 index tumors, tumor still grew quickly after chemotherapy (**Figure** **3**A,C). In contrast, mice bearing with a low sGRP78 index tumors were more sensitive to chemotherapy (Figure 3B,C). In addition to primary tumors, lung metastases were also insensitive to chemotherapy in sGRP78^hi^ MMTV‐PyMT mice (Figure 3D). For sGRP78^lo^ MMTV‐PyMT mice, chemotherapy induced a significant decrease of tumor ^18^F‐FDG uptake, suggesting low aggressiveness of the tumors (Figure 3E).

After verifying the association of chemotherapy response with sGRP78 level, next, to assess the effect of sGRP78, the pair of E0771 cell lines was compared for their chemotherapy sensitivity. Given that E0771(GRP78‐mCherry) group released more sGRP78 than E0771(mCherry) group in vitro (Figure 2A–C) and in vivo (Figure 3F), it was found that E0771(GRP78‐mCherry) tumors shrank more slowly than E0771(mCherry) when treated with nab‐paclitaxel (Figure 3G). Their metastatic potential to peripheral blood (Figure 3H,I) and regional lymph nodes (Figure 3J) was significantly enhanced, as FCM analysis showing that there were 0.1%–0.3% mCherry^+^ tumor cells still present in the blood and LNs even after chemotherapy. Therefore, tumor‐releasing sGRP78 did mediate cancer resistance to chemotherapy.

### 2.4 sGRP78‐Conditioned B Cells Possess Regulatory Function

**Figure 4:**
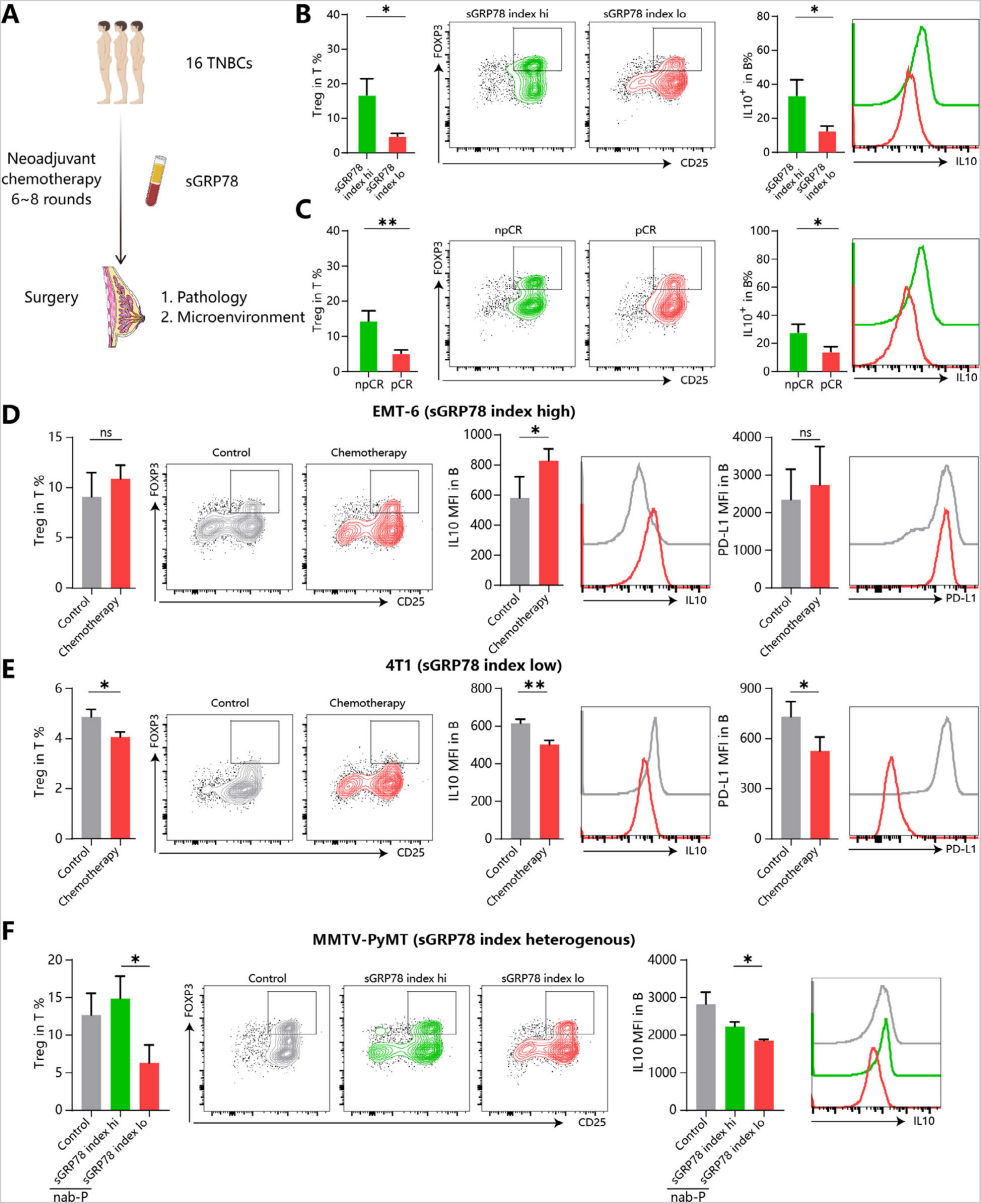
sGRP78 is involved in tumor microenvironmental regulation A) 16 TNBC patients with NAT were included and classified based on their sGRP78 index as sGRP78^hi^ and sGRP78^lo^ patients, and on pathological results post chemotherapy as non‐pCR and pCR patients. The frequencies of tumor‐infiltrating Treg and IL10^+^ Breg in B) sGRP78^hi^ and sGRP78^lo^ patients, C) non‐pCR and pCR patients. D–F) The frequencies of Treg and mean fluorescence intensity of IL‐10, PD‐L1 in B cells in D) EMT‐6 tumor bearing mice with nab‐P (10 mg kg^−1^ i.p. q2d, *n* = 8), E) 4T1 tumor bearing mice treated with Lb (10 mg kg^−1^ i.p. q2d, *n* = 8), and F) MMTV‐PyMT mice treated with nab‐P (20 mg kg^−1^ i.p. q1w, *n* = 8). D–F) MMTV‐PyMT mice were dichotomously grouped according to their sGRP78 index with 4200 ng mL^−1^ as the threshold. ^*^ *p* < 0.05, ^**^ *p* < 0.01. q1w: once per week; q2d: once per two days; i.p.: intraperitoneal injection.

To explore the underlying mechanisms on how sGRP78 confers the resistance, post‐NAT surgical specimens from TNBC patients’ cohort stated above were collected (Table S1, Supporting Information) for analyzing the status of tumor‐infiltrating lymphocytes (**Figure** **4**A). 16 patients were included and dichotomously classified as sGRP78^hi^ or sGRP78^lo^ groups according to their sGRP78 index. It was demonstrated that in sGRP78^hi^ TNBC patients, more Tregs and regulatory IL‐10^+^ B cells infiltrated into tumor tissues (Figure 4B). And markers of exhaustion (PD‐1), death (Fas), naive (CD45RA) were enhanced in infiltrated T cells, as well as the increased frequency of IgA^+^ plasma and impaired expression of Granzyme B in NK cells (Figure S3A, Supporting Information). To understand which cell subsets and immune molecules contributed the most to the sGRP78‐related cancer drug resistance, the above alterations were checked between non‐pCR and pCR TNBC patients. It was observed that npCR patients also exhibited more infiltration of Tregs, IL‐10^+^ B cells, and IgA^+^ plasma cells (Figure 4C; Figure S3B, Supporting Information). In the three murine models, this phenomenon was reproduced in sGRP78^hi^ groups. As shown in Figure 4D–F, EMT6 tumors (sGRP78^hi^) retained more infiltration of Tregs and IL‐10⁺ B cells after chemotherapy than control mice, while in sGRP78^lo^ 4T1 tumors, the frequencies of regulatory lymphocytes were sharply reduced. Similarly, within the MMTV‐PyMT cohort, sGRP78^hi^ tumors harbored more Tregs and IL‐10⁺ B cells than their sGRP78^lo^ counterparts (Figure 4F; Figure S3C, Supporting Information). These data support the conclusion that sGRP78 is involved in the tumor microenvironment regulation after chemotherapy.

**Figure 5:**
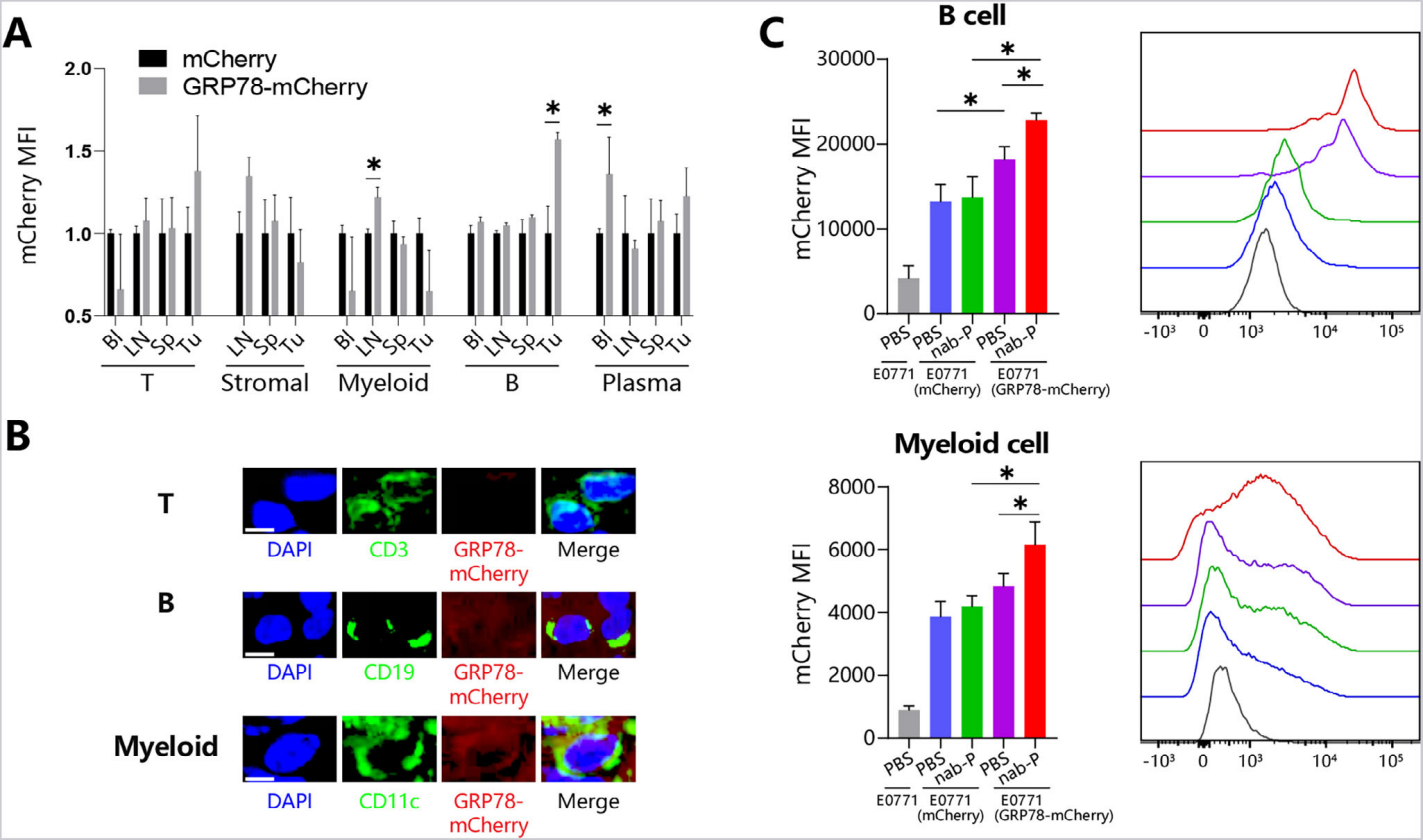
sGRP78 binds with B cells A) The mean mCherry intensity on multiple cell populations isolated from peripheral blood (Bl), lymph node (LN), spleen (Sp), and tumor (Tu) in E0771 (GRP78‐mCherry or mCherry) tumor bearing mice. B) Representative images for GRP78‐mCherry binding with tumor infiltrating CD3^+^ T cells, CD20^+^ B cell or CD11c^+^ myeloid cells from E0771 (GRP78‐mCherry) bearing mice, Scale bar = 5 µm. C) mCherry intensity on tumor infiltrating B cells and myeloid cells in E0771 (wildtype, mcherry or GRP8‐mCherry overexpression) tumor bearing mice treated with nab‐P (10 mg kg^−1^ i.p. q2d, *n* = 8). ^*^ *p* < 0.05.

In the following experiments, to identify the effector cells of sGRP78, T, B lymphocytes, and stromal, myeloid cells were isolated from multiple tissues (including tumor, blood, lymph node, and spleen) of E0771 (mCherry or GRP78‐mCherry) tumor bearing mice and were tested for their mCherry‐emitting capacities. FCM analysis demonstrated that tumor‐infiltrating B cells in GRP78‐mCherry mice emitted more fluorescence than in control mice, as well as myeloid cells, but not T cells (**Figure** **5**A). IHC staining verified the direct binding of B cells with GRP78‐mCherry (Figure 5B). In addition, after chemotherapy, more fluorescence was emitted by B and myeloid cells in GRP78‐mCherry mice than in control mice, confirming that chemotherapy‐induced tumor‐derived sGRP78 did bind with B and myeloid cells in vivo (Figure 5C).

**Figure 6:**
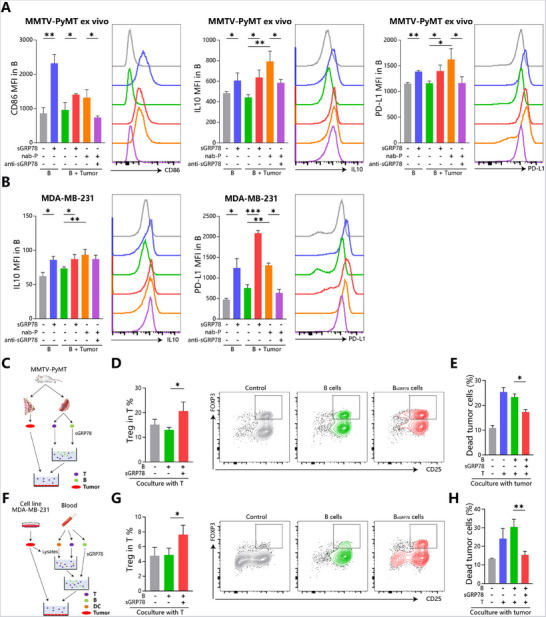
sGRP78‐conditioned B cells suppress the cytotoxicity of T cells A) MMTV‐PyMT splenic B cells were conditioned with sGRP78 (20 µg mL^−1^) or cocultured with cancer cells from the same donor in the presence of nab‐P (10 µm) w/o sGRP78 antagonistic antibody for 3 days. B cell phenotype was analyzed by FCM. B) Expression of IL10, PD‐L1 in human peripheral blood B cells cocultured with MDA‐MB‐231 as above. C–E) Schematic design for investigating the effect of Bgrp78 cells on mice T cells. MMTV‐PyMT splenic B cells were conditioned with sGRP78 (20 µg mL^−1^) for 3 days, and then cocultured with T cells for 7 days. D) Frequencies of CD25^+^FOXP3^+^ Tregs were measured. E) CD8^+^ T cells were isolated to perform cytotoxicity assays for dead tumor cells preloaded with CellTrace. F–H) Schematic design for investigating the effect of Bgrp78 cells on human T cells. Human dendritic cells (DC) were induced from blood monocytes, pulsed with MDA‐MB‐231 lysates as antigen, and primed T cells. These tumor‐specific T cells were cocultured with Bgrp78 cells for 6 days and then evaluated G) Proportions of Tregs and H) dead tumor cells. ^*^ *p* < 0.05, ^**^ *p* < 0.01, ^***^ *p* < 0.001.

Combined with data in Figures 4 and 5, it was speculated that B cells would be one of the targets of sGRP78 to regulate the tumor microenvironment. To verify the hypothesis, B cells were conditioned with sGRP78 or cocultured with cognate tumor cells, followed by detection for immunoregulatory molecules. Data showed that sGRP78‐conditioned B cells upregulated their IL‐10 and PD‐L1 expressions, as well as B cells cocultured with drug‐treated tumor cells. And this upregulation could be blunted by anti‐GRP78 blocking antibody (**Figure** **6**A,B).

Taken together, above results suggest that sGRP78 could convert B cells to the regulatory phenotype.

### 2.5 Adoptive Transfer of sGRP78‐Conditioned B Cells Exacerbates Tumor Immunosuppression

To explore the effect of sGRP78‐conditioned B (Bgrp78) cells on antitumor immunity, cognate T cells were cocultured with them (Figure 6C,F) and then assayed for their cytotoxicity and Treg frequency. Results manifested that after being treated by Bgrp78 cells, T cell populations significantly increased the proportion of Tregs (Figure 6D,G) with attenuated cytotoxicity to MDA‐MB‐231 cells (Figure 6E,H).

**Figure 7:**
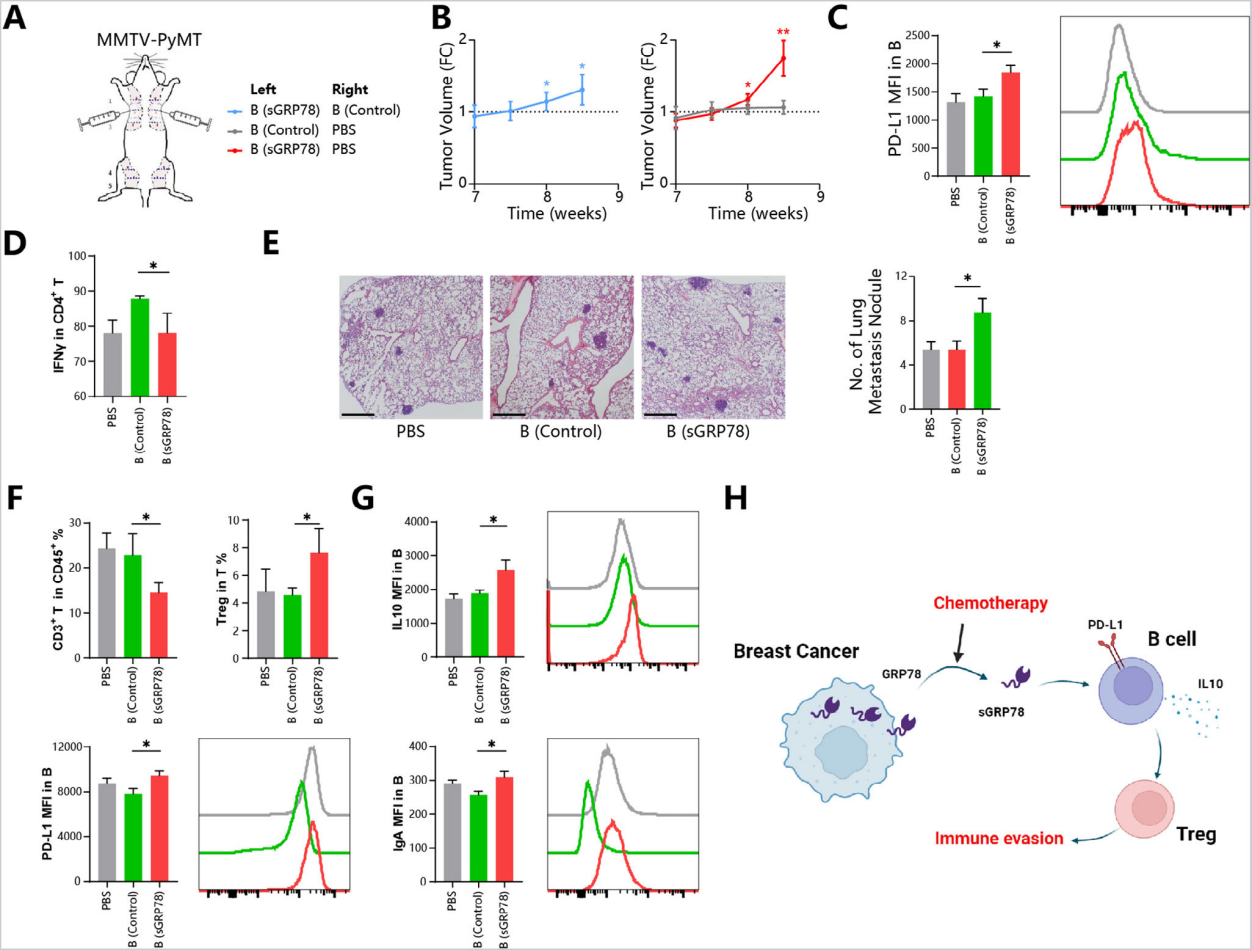
Adoptive transfer of sGRP78‐conditioned B cells facilitates tumor evasion A) Bgrp78 cells and control cells/PBS (2 × 10^6^ cells/50 µL per mouse, *n* = 8) were intratumorally injected into bilateral mammary pads of MMTV‐PyMT strain, respectively. B) Fold change in the tumor volume of the B cell‐treated left pad to the control right pad. C) PD‐L1 expression on tumor infiltrating B cells. D) IFNγ expression on tumor‐infiltrating CD4^+^ T cells. (E,F) MMTV‐PyMT was intravenously injected with sGRP78‐conditioned B cells (3 × 10^6^ cells/100 µL per mouse, *n* = 8). E) The lung metastatic nodules were stained with HE (left) and quantified (right), Scale bar = 500 µm. F) The frequencies of T and Treg. G) Mean fluorescence intensity of IL‐10, PD‐L1, and IgA in B cells. H) Schematic role of tumor‐derived sGRP78 on immune cells. sGRP78 mediates immune evasion by promoting the expression of IL‐10 and PD‐L1 in B cells to induce the formation of Treg cells. ^*^ *p* < 0.05, ^**^ *p* < 0.01.

For MMTV‐PyMT mice, Bgrp78 cells would exacerbate the immunosuppressive tumor microenvironment because more Tregs were induced in T cell populations after co‐cultured with Bgrp78 cells in vitro (Figure 6D). By this, mice were injected with Bgrp78 cells intratumorally and then were assessed for their tumor progression (**Figure** **7**A). Compared with PBS and untreated controls, Bgrp78 cell injection resulted in an enhanced tumor progression (Figure 7B), PD‐L1 upregulation on intratumoral B cells and less tumor infiltration of effector CD4^+^ T cells (Figure 7C,D). Furthermore, intravenous injection of Bgrp78 cells resulted in promoted lung metastases of breast cancers (Figure 7E) and exacerbation of the suppressive lung microenvironment (Figure 7F,G). In conclusion, these in vitro and in vivo data suggest that sGRP78‐conditioned B cells exacerbate the immunosuppression tumor microenvironment, mediating immune evasion (Figure 7H).

### 2.6 Nanobody‐subA Fusions Enable Targeted sGRP78 Degradation

**Figure 8:**
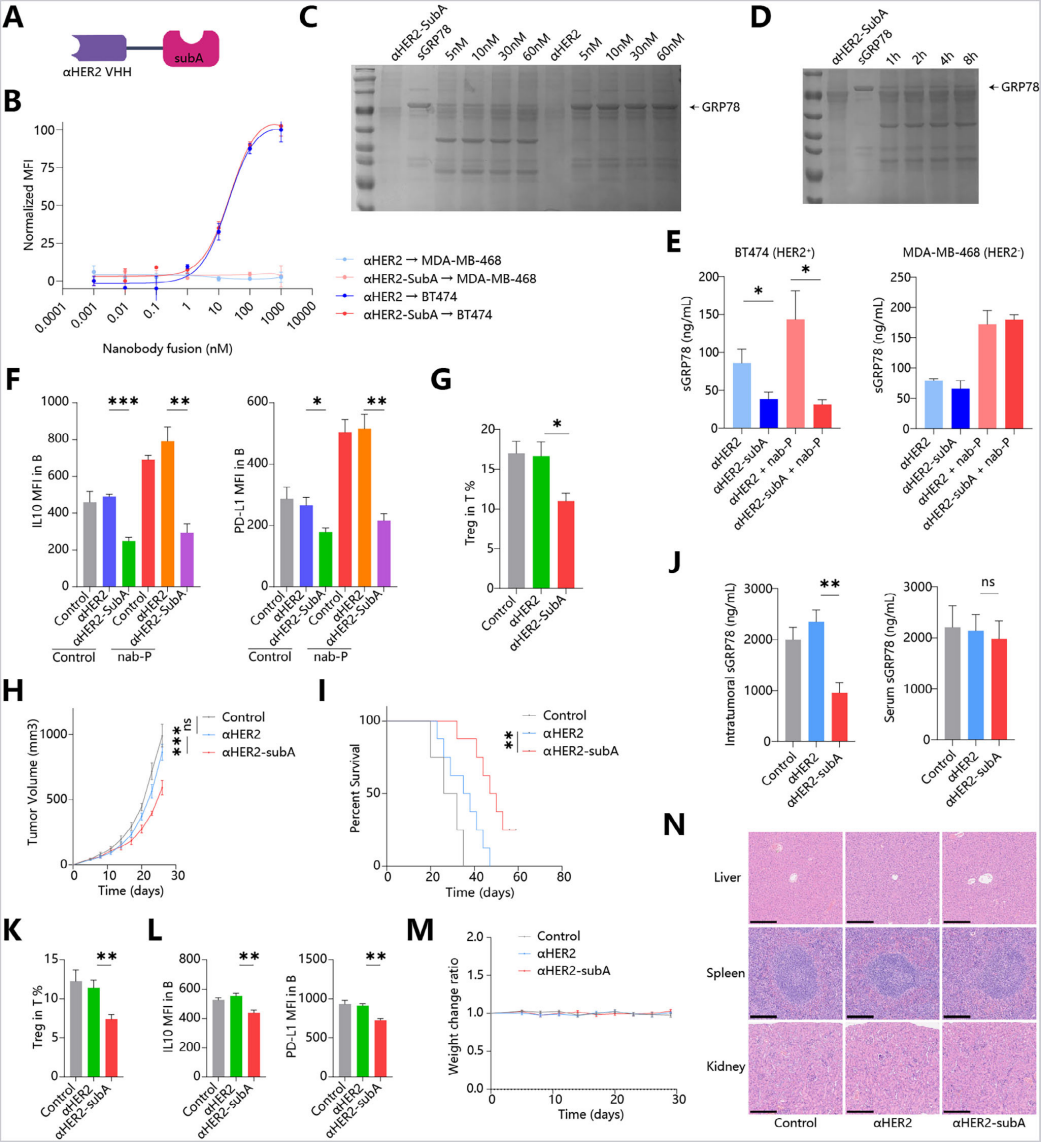
Targeted degradation of sGRP78 by Nanobody‐subA A) Schematic representation of αHER2‐subA. B) Binding levels of αHER2‐subA and αHER2 to HER2^+^ BT474 cells and HER2*^−^* MDA‐MB‐468 cells. C,D) Coomassie Brilliant Blue staining for degradation of sGRP78 C) by αHER2‐subA or αHER2 at indicated concentrations for 12 h and D) by αHER2‐subA(5 nm)for indicated time periods. E) HER2^+^ BT474 cells and HER2^−^ MDA‐MB‐468 cells were incubated with αHER2‐subA or αHER2, then treated with nab‐P. sGRP78 levels in supernatant were detected by ELISA. F) Phenotype of B cells co‐cultured with BT474 cells plus therapeutics. G) Proportion of Tregs in T cells co‐incubated with B cells in (F). H–L) Mice were inoculated with human HER2‐expressing 4T1 cells, followed by treatment with nab‐P and αHER2‐subA or αHER2 (10 mg kg^−1^). Tumor size H), survival I), and sGRP78 levels in tumors and serum J) were measured. K) Frequencies of tumor‐infiltrating Tregs and L) phenotype of B cell. M–N) Healthy mice were injected with nab‐P and αHER2‐subA or αHER2, followed by measurement of body weight M) and histopathological damage, Scale bar = 500 µm (N). ^*^ *p *< 0.05, ^**^ *p *< 0.01, ^***^ *p *< 0.001.

Bacteria‐derived serine proteinase subtilase cytotoxin catalytic A subunit (subA) was reported to selectively degrade GRP78.^[^ ^21^, ^22^ ^]^ We previously verified that recombinant SubA could degrade sGRP78 both in vitro and in vivo.^[^ ^17^, ^23^ ^]^ However, recombinant SubA lacks tissue specificity, which could result in severe adverse effects. To address this limitation, we aimed to develop a drug capable of directly degrading intratumoral sGRP78. To achieve this, a genetic fusion of SubA enzymes and antibodies targeting a cell‐surface receptor specific to breast cancer was designed (**Figure** **8**A). HER2 was selected as the target antigen based on its relevance in breast cancer and its validation in previous FDA‐approved therapies. We prepared conjugates with SubA fused to the C‐terminus of a HER2 nanobody, termed “αHER2‐SubA” (Figure 8A). αHER2‐SubA exhibited a high affinity for HER2‐expressing cancer cells (Kd = 21.5 nM), comparable to that of αHER2 alone (Kd = 19.8 nM), indicating its potential for effective targeting of breast cancer cells (Figure 8B). in vitro experiments demonstrated that αHER2‐SubA efficiently degraded sGRP78 at low concentrations and within short incubation times (Figure 8C,D). Furthermore, ELISA assay confirmed that αHER2‐SubA significantly reduced supernatant sGRP78 levels in HER2^+^ cancer cells but not in HER2^−^ ones (Figure 8E). These findings demonstrate that nanobody‐SubA fusions can specifically target cancer cells to degrade sGRP78.

Next, we tested whether αHER2‐SubA could selectively reverse sGRP78‐dependent induction of regulatory B cells. HER2^+^ breast cancer cells were cocultured with B cells plus nab‐P and αHER2‐SubA or αHER2. It was observed that, regardless of the supplement of chemotherapeutics, αHER2‐SubA can downregulate the expression of IL10 and PD‐L1 in B cells (Figure 8F). These αHER2‐SubA‐conditioned B cells also induced less Tregs formation compared with αHER2 or vehicle groups (Figure 8G). Therefore, these results suggested nanobody‐SubA could degrade cancer‐derived sGRP78 to reverse B cells‐induced CTL dysfunction.

To assess the on‐target efficacy of αHER2‐SubA in vivo, human HER2‐expressing 4T1 cells were orthotopically implanted into the mammary fat pads of mice, followed by treatment with nab‐paclitaxel plus αHER2‐SubA, αHER2 or vehicle control. Data manifested that compared with αHER2 or vehicle, αHER2‐SubA markedly curtailed tumor growth and significantly extended overall survival (Figure 8H,I), with a significant reduction of intratumoral sGRP78. Interestingly, it was found that αHER2‐SubA administration had minimal effect on serum sGRP78, suggesting that αHER2‐SubA preferentially targeted intratumoral sGRP78 for degradation in vivo (Figure 8J). When profiling the tumor microenvironment, the decreased infiltration of Treg (Figure 8K), downregulation of IL‐10 and PD‐L1 in B cells (Figure 8L) were observed as expected.

These data confirm that αHER2‐SubA degrades sGRP78 to reverse B cells‐mediated immunosuppressive tumor microenvironment.

In addition, mice treated with αHER2‐SubA did not exhibit weight loss over the course of the experiment (Figure 8M), and off‐target toxicity was not found in liver, spleen, brain, kidney, heart, and lung (Figure 8N). These biosafety assessments suggested a good tolerance of mice to αHER2‐SubA, assuring its future application in cancer therapy.

## 3 Discussion

The medical community has been committed to finding biomarkers for early screening and prognostic prediction of tumors. In recent years, serum biomarkers have gradually been developed, including proteins, autoantibodies, non‐coding RNAs, methylation, and metabolites.^[^ ^24^ ^]^ Based on clinical cohorts, it was found that some serum proteins can be used to diagnose breast cancer, such as CA15‐3, cytokeratin and human epididymis 4.^[^ ^25^, ^26^ ^]^ In this study, we reported that serum sGRP78 in breast cancer patients was much higher than in healthy people, regardless of the molecular subtype and previous treatment history, indicating that sGRP78 has high sensitivity as an early diagnostic marker for breast cancer. However, the specificity might be low due to induction of GRP78 in other stress settings, such as pulmonary arterial hypertension,^[^ ^7^ ^]^ rheumatoid arthritis,^[^ ^27^, ^28^, ^29^ ^]^ obesity and metabolic syndrome,^[^ ^30^, ^31^ ^]^ as well as other types of tumors.^[^ ^5^ ^]^ In our investigation, it was also observed that serum sGRP78 was significantly elevated in breast cancer patients receiving neoadjuvant therapy, especially after 4th round of chemotherapy (quantified as “sGRP78 index”). And the high sGRP78 index marked the prelude of poor therapy response, recommending that the regimen should be changed. These phenomena suggest that beyond for early diagnosis, sGRP78 could also be identified as a good serological indicator that predicts chemotherapy efficacy. Serum sGRP78 measurement has the advantages of non‐invasiveness, non‐radiation, low cost, and dynamic monitoring, comparing with other current methods, such as imaging, pathology, or circulating tumor cells. As for the choice of serum sGRP78 levels after the fourth round of chemotherapy as the reference point, although the level after the fifth round of chemotherapy exhibited the largest difference between the two groups, 4th round is preferable to 5th for clinical concerns. First, after four rounds of chemo, the radiographic and laboratory assessments would be performed to constitute an interim response evaluation, which directly influences the decision to continue the current regimen or to switch to an alternative neoadjuvant strategy. Hence, 4th round is a good timing for sGRP78 index to be integrated into interim efficacy evaluation. Second, 4th round permits earlier determination of therapeutic efficacy and thus facilitates a more timely therapeutic adaptation. In summary, serum sGRP78 could be used as a biomarker for early diagnosis and chemotherapy response monitoring.

Chemotherapy induces tumor cells to release sGRP78 to the extracellular environment with several possible mechanisms. First, the KDEL motif assists GRP78 located in the endoplasmic reticulum as a molecular chaperone. GRP78 expression significantly increases with KDEL motifs glycosylation after ER stress. These GRP78s will be delivered to the Golgi apparatus with COPII vesicles and then be released in the form of secretory vesicles.^[^ ^32^, ^33^ ^]^ Second, sGRP78 can also be secreted along with tumor‐released exosomes.^[^ ^34^ ^]^ Third, we suggest that tumor cell membranes would be the major source of sGRP78 because chemotherapeutics treatment could quickly reduce the GRP78 level on the cell membrane while elevating its serum level. Due to the complex mechanisms, sGRP78 release is influenced by various factors, including molecular phenotype of tumor cells, sensitivity to chemotherapy drugs, and treatment course. Therefore, there are significant individual differences in the circulating sGRP78 level, which makes sGRP78 a highly discriminative biomarker for clinical use.

The immunoregulatory property of sGRP78 was proposed by VM Corrigall et al.^[^ ^7^ ^]^ We also reported that sGRP78 bound to CD14 on DCs and macrophages, thereby inducing TLR4 endocytosis and degradation, blocking the downstream signaling pathway, including reduced release of pro‐inflammatory cytokines (IFNβ, IL‐1β, IL‐6, and TNFα),^[^ ^11^ ^]^ induction of DCs to a tolerant phenotype,^[^ ^10^ ^]^ M2 polarization of macrophages.^[^ ^16^ ^]^ These sGRP78‐conditioned myeloid cells could affect the activation of liver NK cells to promote breast cancer liver metastasis.^[^ ^16^ ^]^ Besides myeloid cells, our preliminary data supported that B cells were another sGRP78 target that could be modulated into regulatory ones in vitro.^[^ ^9^ ^]^ This study confirmed that chemotherapy‐induced sGRP78 bound with both CD11c^+^ myeloid cells and CD19^+^ B cells derived from primary breast cancer. And B cells were found to evidently increase their expression of IL‐10, PD‐L1 in sGRP78^hi^ breast cancer samples from patients and mouse models. Thence, our study proposes that sGRP78, as a novel regulatory factor of the tumor microenvironment after chemotherapy, directly affects the phenotype of tumor‐infiltrating B cells.

In recent years, the role of B cells in the tumor microenvironment has received increasing attention.^[^ ^35^, ^36^ ^]^ As an integral component of the tumor microenvironment, tumor‐infiltrating B lymphocytes (TIBs) exist in all stages of cancer and play important roles in shaping tumor development. For instance, TIBs can be observed in all stages of human lung cancer development, and their presence differs between stages and histological subtypes.^[^ ^37^ ^]^ In breast cancer, the frequencies of CD19^+^‐IL‐10^+^ B reg cells correlated with shorter OS, and the coexistence of B reg cells with regulatory T cells correlated with shorter metastasis‐free survival.^[^ ^38^ ^]^ TIBs consist of different subpopulations with opposite functions. They switch their pro‐ or anti‐tumor phenotype in different tumor microenvironment or upon chemotherapy.^[^ ^39^ ^]^ It was reported that oxaliplatin induced intratumoral IgA^+^PD‐L1^+^IL‐10^+^ B cell infiltration in prostate cancer and inhibited T cell activation, thereby mediating immune evasion after chemotherapy.^[^ ^40^ ^]^ Neoadjuvant chemotherapy activated the complement signal from apoptotic tumor cells, which induced GRP78^+^ B cells emerging, thereby strengthening the immune function of T cells in breast cancer.^[^ ^39^ ^]^ In addition to the complement signal, we reported here that another signaling molecule, sGRP78, can be induced by chemotherapy and modulate B cells into a distinct IL10^+^PD‐L1^+^ B subset, inhibiting anti‐tumor immunity. This study revealed the complex regulation of the immune microenvironment by chemotherapy, which may have different effects in different individuals.

In summary, this study reveals that chemotherapy significantly induces sGRP78 release from breast cancer cells and profoundly re‐shapes the plasticity of B cells to exacerbate the immunosuppressive tumor microenvironment. Equally importantly, we propose sGRP78 has potential as a predictive serological marker and a therapeutic target to enhance the efficacy of immunogenic chemotherapy in breast cancer.

## 4 Experimental Section

### Patient Data and Study Approval

Serum samples from two cohorts were obtained from Union Hospital, Tongji Medical College, Huazhong University of Science and Technology, according to IRB‐approved protocols with informed consent. The first cohort includes 14 healthy volunteers and 66 breast cancer patients who were newly diagnosed or had already received chemotherapy. The sera were collected once for each donor. The second cohort includes 43 breast cancer patients who were determined to undergo NAT based on the clinical guide and finally received surgery. The serum before each round of chemotherapy and before surgery were collected. Tumor samples were collected according to a standard pathology procedure. The NAT regimens were as follows: doxorubicin 60 mg m^−2^ plus cyclophosphamide 600 mg m^−2^ every three weeks for four cycles, followed by paclitaxel (80 mg m^−2^) for 12 weeks. HER2‐positive patients were treated with Trastuzumab and/or Pertuzumab. Therapeutic effects were evaluated according to the standard of Response Evaluation Criteria in Solid Tumors (RECIST). Pathological complete response (pCR) was defined as no residual invasive carcinoma or ductal carcinoma in situ (DCIS) in any excised breast or lymph node tissue after NAT. Serum samples from 14 healthy volunteers were obtained as controls.

### Mice

C57BL/6, BALB/c mice were purchased from HFK Bioscience (Beijing, China), and MMTV‐PyMT mice from Jackson Laboratory (Bar Harbor, USA). They were kept in pathogen‐free conditions. Female mice were utilized at 6–8 weeks of age. Mice were randomized at the beginning of each experiment, and experiments were not blinded.

### Cell Culture and Chemotherapeutic Reagents

All cells were obtained from the American Type Culture Collection, and grown in a 37 °C incubator with 5% CO2. Breast cancer cell lines E0771, 4T1, and EMT‐6 were cultured in DMEM (Gibco) supplemented with 10% fetal bovine serum. The cells tested negative for mycoplasma contamination and were authenticated by short tandem repeat profiling before use. E0771 cells were stably transfected with the PB transposon system containing a CMV promoter‐driven GRP78‐mCherry coding sequence to obtain E0771 (GRP78‐mCherry) cells.^[^ ^16^ ^]^ 4T1, EMT‐6 and B16‐F10 were treated the same to obtain GRP78‐mCherry overexpressing cell lines. Pemetrexed (Qilu pharma), nab‐Paclitaxel (nab‐P, Soyum pharma), Cyclophosphamide (Hengrui Medicine), Etoposide (Qilu pharma), Lobaplatin (Lb, Changan pharma), Vindesine (Yangtze River Pharm), Docetaxel (Sanofi), Methotrexate (Pude pharma), Decitabine (Qilu pharma), Cytarabine (Pfizer), Doxorubicin (SOYUM pharma), Carboplatin (Qilu pharma), Dacarbazine (Lingnan pharma), Bleomycin (Hanhui pharma), and anti‐GRP78 blocking antibody were supplemented into the culture media as needed.

### Isolation of Immune Cells

Human peripheral blood mononuclear cells (PBMCs) were isolated from whole blood by density gradient centrifugation. Then, T and B cells were magnetically purified using Stemcell EasySep Human T Cell Isolation Kit and B Cell Isolation Kit, and CD14^+^ monocytes by CD14 MicroBeads (Miltenyi Biotec). Monocyte‐derived dendritic cells (moDCs) were obtained as previously reported.^[^ ^41^ ^]^ Briefly, CD14^+^ monocytes were cultured in 1640 medium supplemented with GM‐CSF (800U mL^−1^; R&D) and IL‐4 (400U mL^−1^; R&D) for 7 days to generate moDCs. Mouse splenocytes and tumor suspension were prepared, and then T and B cells were magnetically purified using Stemcell Isolation Kit, respectively.

### Preparation of Recombinant Proteins

Recombinant mouse GRP78 was prepared as described previously.^[^ ^11^, ^17^ ^]^ Nanobody and nanobody‐SubA fusions were expressed and purified from Expi293 cells using transient transfection (Expifectamine, Thermo Fisher Scientific). Enhancers were added 20 h after transfection. Cells were incubated for 5d at 37 °C and 8% CO2. Medium was then collected by centrifugation at 4000 g for 20 min. Proteins were purified by Ni‐NTA affinity chromatography, buffer exchanged into PBS containing 20% glycerol, concentrated, and flash frozen for storage at −80 °C. Purity and integrity of all proteins were assessed by SDS–PAGE. Pre‐ and post‐freeze stability was assessed via UV‐vis spectrophotometry as well as SDS–PAGE.

### Flow Cytometry

Suspended cells were stained for Fixable Viability Stain 510 or 780 (BD Horizon) in PBS for 30 min at 4 °C to distinguish live and dead cells. To block non‐specific binding, mouse and human cells were incubated with FcR blocking reagent for mice and humans (BioLegend), respectively. Cells were stained with fluorescent dye conjugated antibodies, including anti‐mouse CD3 (BD; 17A2), CD4 (BioLegend; RM4‐5), CD8 (BioLegend; 53–6.7), CD19 (BD; 1D3), CD25 (BD; M‐A251), CD27 (BioLegend; LG.3A10), CD45 (BD; A20), CD86 (BioLegend; GL‐1), CD138 (BioLegend; MI15), EpCAM (BioLegend; G8.8), GRP78 (CST; C50B12), IgA (eBioscience; mA‐6E1), IgD (BioLegend; 11–26c.2a), IL10 (BD; JES5‐16E3), PD‐L1 (BioLegend; 10F.9G2), and anti‐human CD3 (BD; SK7), CD4 (BioLegend; RPA‐T4), CD19 (BioLegend; HIB19), CD25 (BioLegend; BC96), CD45 (BioLegend; HI30), and EpCAM (BioLegend; CO17‐1A). For FOXP3 detection, cells were stained using Transcription Factor Staining Set (BD), anti‐human FOXP3 antibody (BD; 236A/E7), or anti‐mouse FOXP3 antibody (Biolegend; MF‐14). For intracellular staining of IL‐10, Cytofix/Cytoperm solution (BD) was added before fixation and permeabilization. All samples were tested by FACSVerse analyzer (BD Biosciences, USA), and data were analyzed using FlowJo v.0.5.3 software (TreeStar).

### Immunofluorescence

Tumor tissue cryosections were stained with antibodies against CD3 (Abcam, Clone SP7, dilution 1/400), CD11c (Proteintech, 17342‐1‐AP, dilution 1/200), CD19 (Abcam, Clone EPR23174‐145, dilution 1/200), and then with AF488‐labeled donkey anti‐rabbit IgG (Jackson ImmunoResearch,711‐546‐152). Nuclei were stained with DAPI (Sigma–Aldrich). Images were acquired with Zeiss LSM880 confocal microscopy and analyzed using ImageJ.

### Immunoblot

Whole‐cell lysates were separated by 12% SDS‐PAGE followed by blotting analysis with anti‐ GRP78 (Abcam, ab32618, dilution 1/1000), ATP1A1 (Proteintech, 55187‐1‐AP, dilution 1/1000), and HRP‐conjugated secondary antibodies (Proteintech, 66009‐1‐Ig, dilution 1/1000). The positive immune reactive signal was detected using an ECL kit (Fude Biotech, Hangzhou, China) and photographed by the ChemiScope Imaging System (Clinx, Shanghai, China). β‐actin was used as an endogenous control.

### sGRP78 Measurement

Concentration of serum sGRP78 was measured using GRP78/BiP ELISA Kit (Enzo) following the manufacturer's recommendations. The fluorescence intensity of GRP78‐mCherry in culture supernatant was measured using BioTek Cytation 3 hybrid multi‐mode microplate reader.

### Co‐Culture Assays

To investigate the effect of sGRP78 on B cells, isolated B cells were cocultured with recombinant GRP78 (20 µg mL^−1^) or tumor cells (B:T = 5:1) in the presence of nab‐paclitaxel (10 µm) w/o sGRP78 antagonistic antibody for 3 days.

To investigate the effect of sGRP78‐conditioned B cells (Bgrp78) on human T cells from the same donor, T cells were primed with tumor antigen‐pulsed moDCs for 6 days and cocultured with Bgrp78 (1:1) for another 2 days. Then CD8^+^ T cells were purified magnetically (Stemcell) and mixed with pre‐stained tumor cells (CellTrackerTM Deep Red Dye, Thermo Fisher Scientific) at an E:T ratio of 10:1 for 12 h. For murine splenic T cells, they were cocultured with Bgrp78 cells for 2 days. Sorted CD8^+^ T cells were then mixed with pre‐stained tumor cells for 12 h. After staining with PI (5 µm), the cell mixture was analyzed by FCM for PI^+^ ones in CellTrace‐loaded cells.

### Cell Surface Binding Analysis

Cells were trypsinized, quenched with media, and transferred to a 96‐well V‐bottom plate. Cells were washed three times with PBS + 0.5% BSA + 5 mm EDTA (FACS buffer) and incubated with nanobody‐subA fusions or nanobodies for 30 min on ice. Cells were washed three times and incubated with 25 nm AF488‐labeled anti‐His. Flow cytometry was performed to evaluate cell surface binding.

### Protein Degradation Analysis

Cells were plated in 6‐ or 12‐well plates and grown to ≈70% confluency before treatment. Medium was aspirated, and cells were treated with nanobody‐subA fusions or nanobodies in complete growth medium. After incubation at 37 °C for the designated amount of time, cells were washed with PBS, lifted with EDTA (Gibco; 15040066), and collected by centrifugation at 300 g for 5 min at 4 °C. Samples were then tested by ELISA to quantify protein levels.

### Mice Models for Breast Cancer

Spontaneous breast cancer formation was displayed in MMTV‐PyMT strain. 4T1 and EMT6 tumor allografts were established by subcutaneously injecting cells into the mammary fat pads of BALB/c mice, and E0771 (GRP78‐mCherry) allografts of C57BL/6 mice. These model mice were administered chemotherapeutics by tail vein or injected with Bgrp78 cells intratumorally. The therapeutic effect was assessed by ^18^F‐FDG PET/CT imaging as in our previous report.^[^ ^42^ ^]^ Mice were sacrificed when the tumor diameter reached 15 mm. Blood, spleen, LNs, and tumors were collected. Tissues were digested with a mixture of 0.5 mg/ml DNase (Sigma‐Aldrich) and 1 mg/ml Collagenase IV (Sigma‐Aldrich) in serum‐free RPMI for 30 min. Then single‐cell suspension was dispersed through a 70 µm filter. Erythrocytes in whole blood and spleen samples were lysed using the BD Pharm Lyse buffer (BD). To assess the in vivo effects of Nanobody‐subA fusions, mice were intravenously injected with purified αHER2, and αHER2‐subA, each at a dose of 10 mg kg^−1^, administered every three days. Animals were monitored daily; tumors were measured as previously described, and mouse weight was assessed throughout the study. Animals were euthanized upon reaching humane endpoints: loss of 20% of body weight, breathing impairment, or poor response to external stimuli. No signs of animal suffering or discomfort were observed during the experiment. For survival monitoring, each mouse was sacrificed individually when tumors reached 1000 mm^3^. Mice were sacrificed when any diameter reached 15 mm. All procedures were approved by the Ethics Committee of Tongji Medical College of Huazhong University of Science and Technology.

### Statistical Analysis

The information about statistical details and methods was indicated in the figure legends, text or methods. The measurements of all statistical values were performed using the R environment (v4.1) and Graphpad Prism 9.0, unless otherwise described. Error bars in the experiments indicate standard error of the mean (SEM) or standard deviation (SD) for a minimum of three independent experiments.

Univariable analyses employed binary logistic regression to assess potential risk factors and calculated odds ratios (ORs) with 95% confidence intervals (CIs) for pCR of neoadjuvant therapy. The rates of ER positive, HER2 positive, Ki67 positive in pre‐NAT sample, sGRP78 index, Lymph node metastasis were evaluated for pCR risk. Variables demonstrating significant associations in univariable analyses were included in subsequent multivariable logistic regression models. All models satisfied multicollinearity assumptions.

Multivariate data were analyzed using one‐way and two‐way ANOVA followed by Tukey's multiple comparisons post hoc tests. Nonparametric data were analyzed by Welch's *t*‐test. Two‐tailed unpaired t‐test and multiple t‐test with FDR correction analyzed comparisons between two conditions. A *p *< 0.05 was considered statistically significant.

## Conflict of Interest

The authors declare no conflict of interest.

## Author Contributions

W.Z.H., Z.P. and X.Y.X. contributed equally to this work. L.P. and H.T. were responsible for the conception and design of the study. P.X., Z.X.Q., L.Y.B. and X.J.Y. contributed to the acquisition of data by providing required samples and clinical information. W.Z.H., Z.P. and X.Y.X. performed the animal and cellular experiments. W.Z.H. and L.P. carried out the analysis and interpretation of data, including statistical and bioinformatics analysis. L.P., H.Y. and W.Z.H. wrote and revised the final manuscript. Study supervision was provided by L.P., H.T. and H.Y.

## Supporting information



Supporting Information

## Data Availability

The data that support the findings of this study are available on request from the corresponding author. The data are not publicly available due to privacy or ethical restrictions.
