# Supplementary material for: Targeted Degradation of sGRP78 Alleviates the Immunosuppressive Tumor Microenvironment
Source: Adv Sci (Weinh). 2025 Sep 11;12(45):e09921. doi: 10.1002/advs.202509921 (PMC12677628; doi:10.1002/advs.202509921)
Supplement: Supplementary file 1 — Supporting Information [file ADVS-12-e09921-s001.docx]

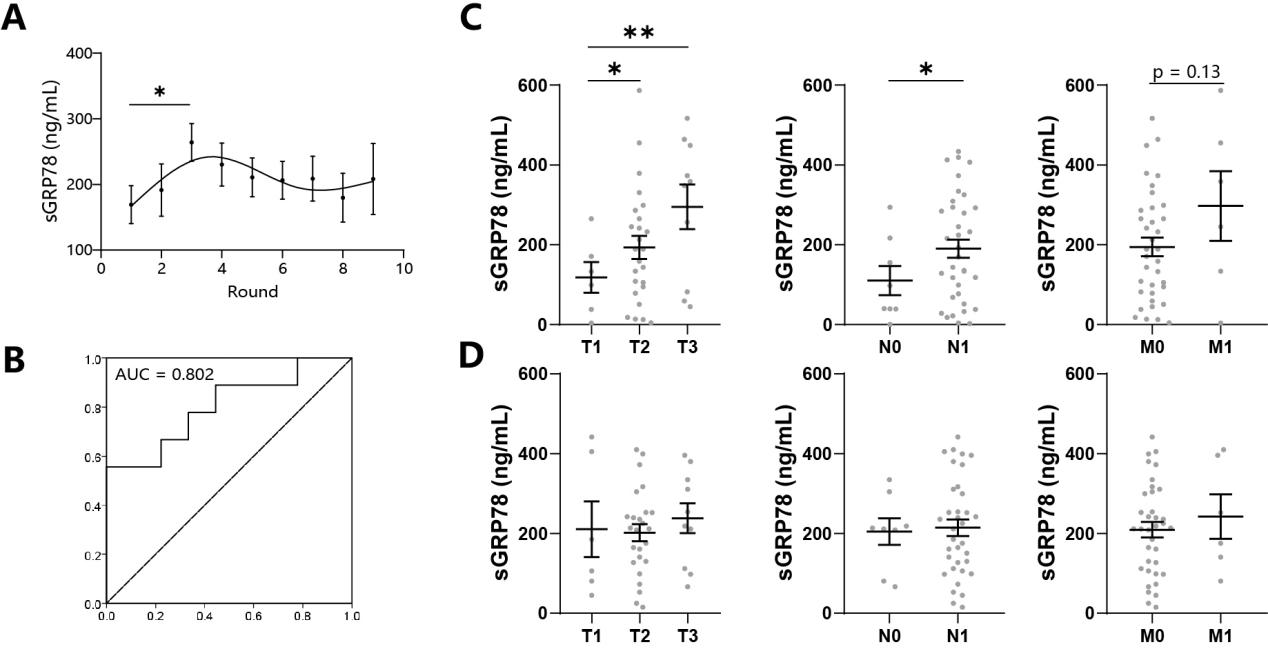


**Fig. S1 sGRP78 release during neoadjuvant chemotherapy**

**(A)** The dynamic changes of serum sGRP78 in breast cancer patients receiving multiple rounds of NAT. **(B)** Receiver operating characteristic (ROC) curve illustrates the ability of sGRP78 index to predict pCR with varied discrimination threshold. **(C, D)** The relationship between tumor T, N, and M stages and serum sGRP78 concentration in **(C)** new-diagnosed patients and **(D)** during NAT. *P<0.05, **P<0.01.


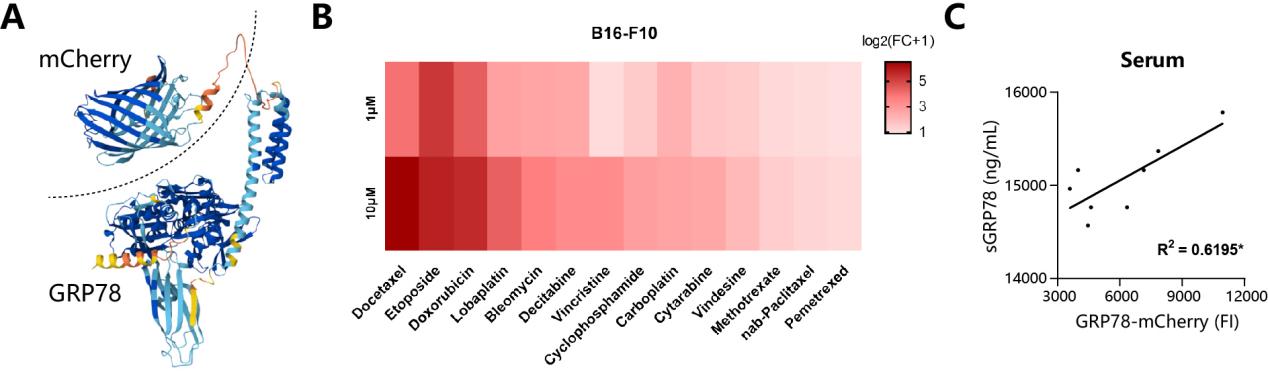


**Fig. S2 Chemotherapy induces cancer cells to release sGRP78**

**(A)** The structure of the GRP78-mCherry fusion protein as predicted by Alphafold is shown, with colors denoting pIDDT levels (>90: dark blue; 70 - 90: sky blue; 50 - 70: yellow; <50: orange). **(B)** Fold change (FC) of GRP78-mCherry fluorescence intensity in the supernatant of B16-F10 (GRP78-mCherry) cells treated with chemotherapeutic drugs (1 or 10 μM) for 48 hours. **(C)** Correlation analysis between the intensity of tumor GRP78-mCherry and serum sGRP78 level in nab-P-treated 4T1(GRP78-mCherry) mice.


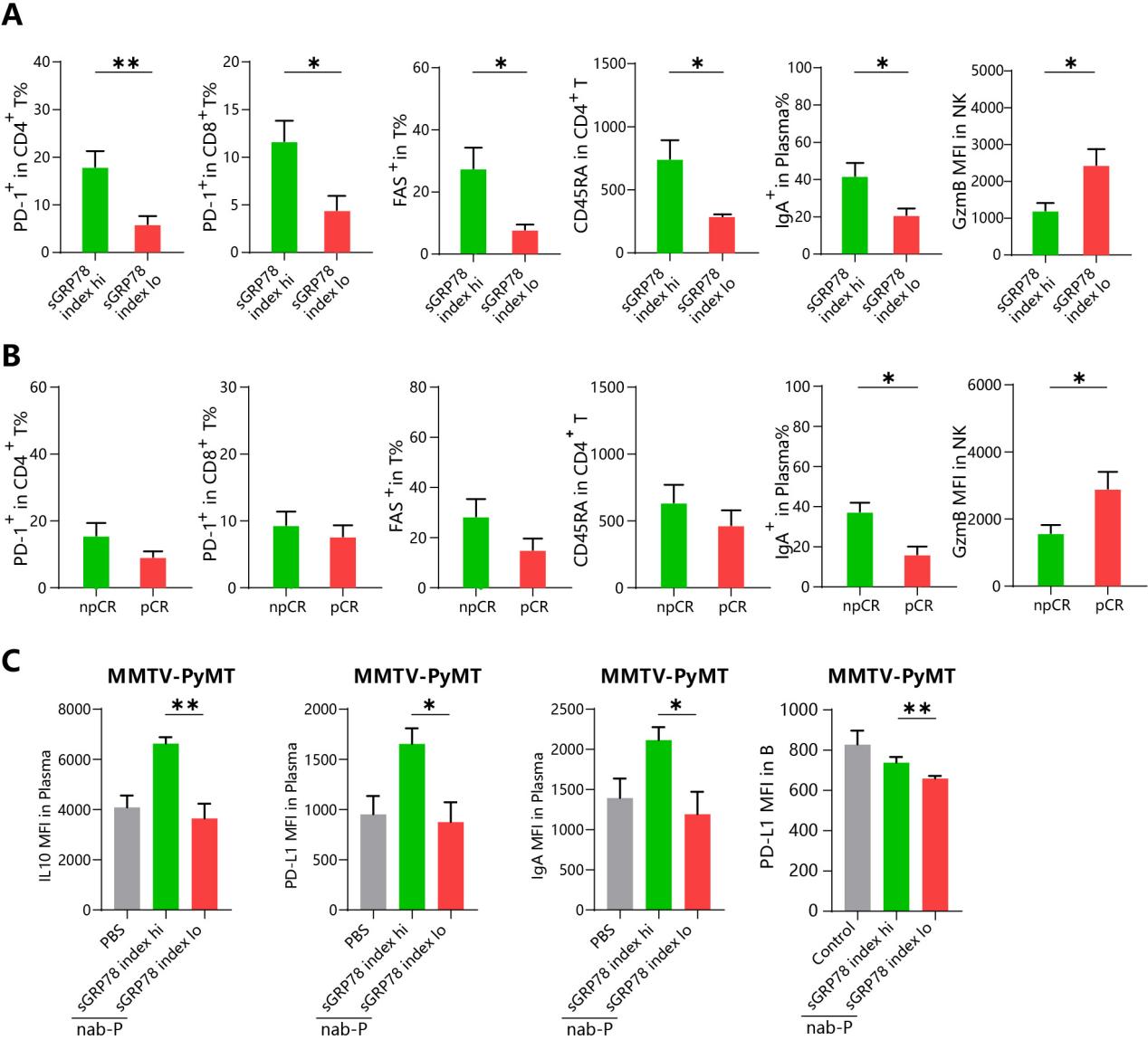


**Fig. S3 sGRP78 is involved tumor microenvironmental regulation after chemotherapy**

Phenotypes of immune cells in **(A)** sGRP78^hi^ and sGRP78^lo^ TNBC patients, **(B)** non-pCR and pCR TNBC patients**. (C)** Phenotypes of B and plasma cells in nab-P treated MMTV-PyMT mice. *P<0.05, **P<0.01.

**
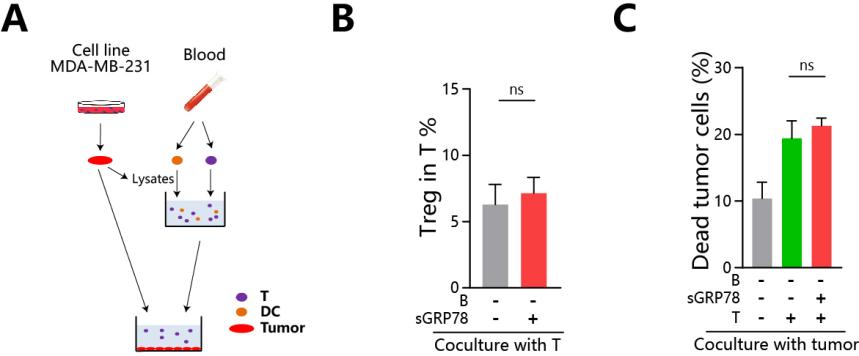
**

**Fig. S4 sGRP78 don’t directly suppress the cytotoxicity of T cells**

(A-C) Schematic design for investigating the effect of sGRP78 on human T cells. Human dendritic cells (DC) were induced from blood monocytes, pulsed with MDA-MB-231 lysates as antigen, and primed T cells. These tumor-specific T cells were treated with sGRP78 for2 days and then evaluated (B) Proportions of Tregs and (C) dead tumor cells.


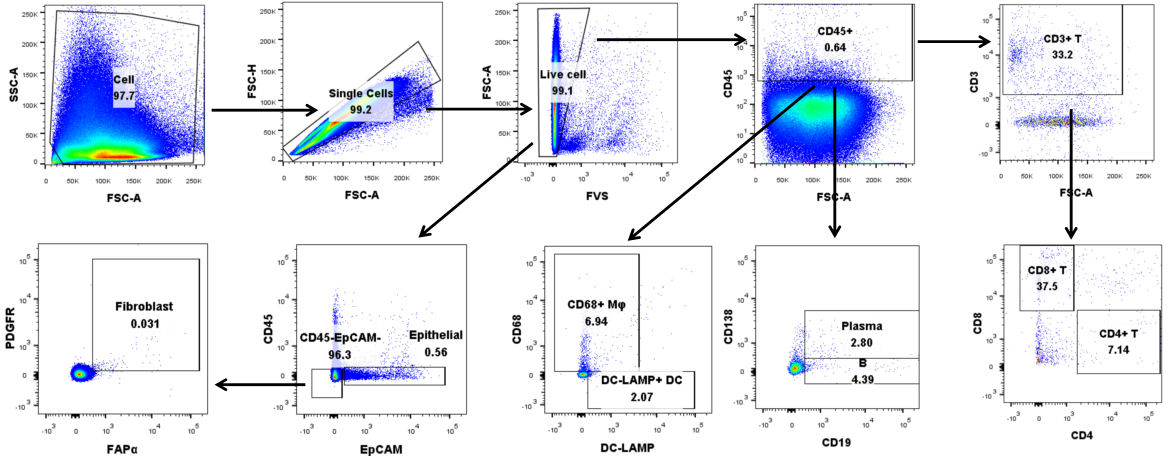


**Fig. S5 Gating Strategy**

Table S1 Clinical features of breast cancer patients

| Clinical features | Number |
| --- | --- |
| **Subtype** |  |
| Luminal A | 1 |
| Luminal B | 10 |
| TNBC | 20 |
| HER2 | 12 |
| **Histological type** |  |
| Invasive ductal carcinoma | 36 |
| Others | 7 |
| **Age** |  |
| <50 | 25 |
| ≥50 | 18 |
| **T stage** |  |
| T1 | 6 |
| T2 | 27 |
| T3 | 10 |
| **LN metastasis** |  |
| Yes | 35 |
| No | 8 |
| **Distant metastasis** |  |
| Yes | 6 |
| No | 37 |
| **Response for neoadjuvant chemotherapy** |  |
| pCR | 15 |
| npCR | 28 |
| **Total** | 43 |

| Table S2. Risk factors associated with non-pathological complete response (npCR) in breast cancer patients with neoadjuvant therapy (NAT) | | | | | | |
| --- | --- | --- | --- | --- | --- | --- |
| Categories | Univariate logistic regression | | | Multivariate logistic regression | | |
|  | OR | 95% CI | P | OR | 95% CI | P |
| ER positvie rate in pre-NAT sample | 3.8 | 1.8~8.0 | 0.001 | 3.2 | 1.1~9.2 | 0.03 |
| HER2 positvie in pre-NAT sample | 1.5 | 1.1~2.1 | 0.02 | 1.9 | 0.6~6.2 | 0.29 |
| Ki67 positvie rate in pre-NAT sample | 7.1 | 2.5~20.2 | 0.0003 | 5.1 | 0.6~44.9 | 0.15 |
| sGRP78 index | 3.6 | 1.7~7.6 | 0.0008 | 2.9 | 1.1~8.1 | 0.04 |
| Lymph node metastasis | 1.6 | 1.2~2.2 | 0.004 | 1.9 | 0.7~5.2 | 0.21 |
